# Artificial Impregnation

**Published:** 1915-05

**Authors:** 


**Artificial Impregnation.** H. Rohleder, Wiener Klin. Rundschau, 1914, No. 22. The technic involves the injection of fresh sperm into the cervix, with a Braun uterine syringe, and the placing of a gauze tampon soaked in sperm over the cervix. The limitation of the method is obvious. By a careful selection of cases, 30% of successes have been achieved.

